# Cultural adaptation and validation of the SPICT-ES™ instrument to identify palliative care needs in Chilean older adults

**DOI:** 10.1186/s12904-022-01111-2

**Published:** 2022-12-16

**Authors:** Ximena Farfán-Zuñiga, Melissa Zimmermann-Vildoso

**Affiliations:** Universidad de los Andes, Chile, Santiago, Chile

**Keywords:** Palliative care, SPICT-ES Instrument, Identification tool, Chilian

## Abstract

**Introduction:**

Chile presents a context of an aging population and increased life expectancy, leading to many older adults (OA) needing palliative care (PC) during the course of their illness. The SPICT-ES™ is an instrument used to clinically evaluate patients with advanced chronic illness (ACI) to detect PC needs. Validating this instrument in Chile will allow for early detection of OA at risk of clinical deterioration who require this care.

**Objective:**

Adapt and validate the SPICT-ES™ instrument to identify PC needs among OA in Chile.

**Methodology:**

Study following quantitative design – cross-sectional, descriptive, and developed in three stages: cultural adaptation by expert judgment; preliminary test of the SPICT-ES^CH^ instrument to evaluate reliability and application of the SPICT-ES^CH^ in 292 patients, to determine internal consistency and stability of the instrument. This study was done between January 2019 and July 2021. Participants in the study were nurses and OA from 5 health centers in Santiago, Chile. This study was approved by the Ethics Committee of Universidad de los Andes.

**Results:**

In the cultural adaptation with content validity, following expert judgment, all items were kept. Semantic modifications were made on only three of them. A Lawshe coefficient of 84% which determined SPICT-ES^CH^ as an acceptable instrument for the following stages of validation and reliability. The pilot for the new version in Chile, SPICT-ES^CH^, determined stability and consistency over time, with a Pearson correlation coefficient (ρ) of 0.9167 (*p* < 0.0001). In the final application of the instrument, to fortify the psychometric evaluation (*n* = 292) we identified 53.4% positive SPICT-ES^CH^. The logistical model via OR (< 0.001) showed that the items predicted the positivity of the instrument. The internal consistency obtained was 0.8662, confirming a correlation and intercorrelation between items. 100% of nurses evaluated the SPICT-ES^CH^ as a useful and feasible instrument.

**Conclusion:**

SPICT-ES^CH^ includes all the relevant indicators for adequate clinical identification of PC needs among the Chilean OA population, who could Benefit from the early introduction of palliative support contributing to their quality of life.

## Introduction

Chile presents a set of challenges aligned with overall global population aging [1]. It is estimated that by 2050, the population aged over 60 will rise by 109.5% [2]. It is the only country in the Latin American region with a life expectancy over 80 years [3]. The accelerated aging of the population, together with the increase in life expectancy, has led to a higher burden of disease and years of life lost due to disability, disease or premature mortality attributable to aging. In this context, Chile is one of the countries with a low level of healthy aging [1] and a high mortality rate (64.5%) in people aged 70 years and older. The first cause of death in Chile, is cancer (28.8%), displacing cardiovascular diseases (26.6%), followed by diabetes and renal diseases (7.8%); digestive diseases (7.45%); neurological disorders (6.06%) and respiratory diseases (5.65%) [4]. There are higher risks of death in OA in lower socioeconomic groups, with less schooling, and these differences are even greater in women (5,6).

This epidemiological context entails an unmet demand in Chile, related to changes in public policies [5] and professional practice regarding patients not only with oncologic disease but also with advanced chronic diseases (ACI), with a disease trajectory perspective, which implies early introduction of palliative support.

The early palliative care is associated with better quality of life [6] and lower organ failure and fragility in end stages [7]. PC needs are not only limited to oncological patients. It is estimated that 75% of the global population will need palliative support due to one or more advanced chronic illnesses (ACI) [8]. It is crucial to have early detection for patients who need PC to fulfill their needs on different personal levels: physical, psychosocial and spiritual [3]. Having validated instruments which can objectify this clinical inflection point, is fundamental for planning treatment at different care levels [9].

The identification of this clinical transition is more intuitive in patients with cancer, due to the course of their disease. However, in patients with ACS, due to their continuous decompensations and clinical situation, life prognosis can be highly variable, which makes it difficult to identify the palliatives needs. The training of professionals and the health care resources allocated to this service in Chile are scarce, so that the availability of clinical instruments for the early identification of these individuals is a challenge for the country [10].

In 2010 the University of Edinburgh Primary Palliative Care Research Group and NHS Lothian (Edinburgh, Scotland) developed the “Supportive & Palliative Care Indicators tool”, SPICT™^.^ It was a tool to identify PC needs and to avoid “prognostic paralysis” [11]. It has been validated in different European countries and translated into Spanish. The SPICT-ES™ version is a quantitative and multifactorial analysis based on general and clinical indicators. It is an easily understood instrument for implementation [10, 11]. An equal cutoff point is established with two general positive indicators and one clinical indicator para determiner a *"SPICT-ES positive",* i.e., a patient who is a candidate for palliative support. Evidence has shown adequate sensitivity and specificity for SPICT-ES™ for identifies PC needs consistently [9, 12].

There is currently a favorable evolutionary scenario PC coverage for non-oncological patients. Specifically, Chile in 2021 passed a law which provides for palliative care and the rights of people suffering from terminal or severe illnesses [13]. The lack of validated instruments which can identify this patient group delays PC provision, giving rise to the objective of this study; The purpose of this study is adapt and validate the SPICT-ES™ instrument to identify PC needs among OA in Chile.

### Hypothesis

The SPICT-ES™, will allow us to reliably and validly identify the need for palliative care among Chilean OA with adequate psychometric properties to be used as a clinical instrument.

## Materials and methods

Quantitative, longitudinal and analytical study design. Study performed between June 2019 and July 2021. For its development, approval was obtained from the Scientific Ethical Committee of the Universidad de los Andes (CEC201835) and the authorization of the author of both the original SPICT™ instrument [14] and its version translated into Spanish, SPICT- ES™, which was used in this study [10]. All methods used in this project were carried out in accordance with national and international guidelines and standards governing research ethics in humans.

**Fig. 1:**
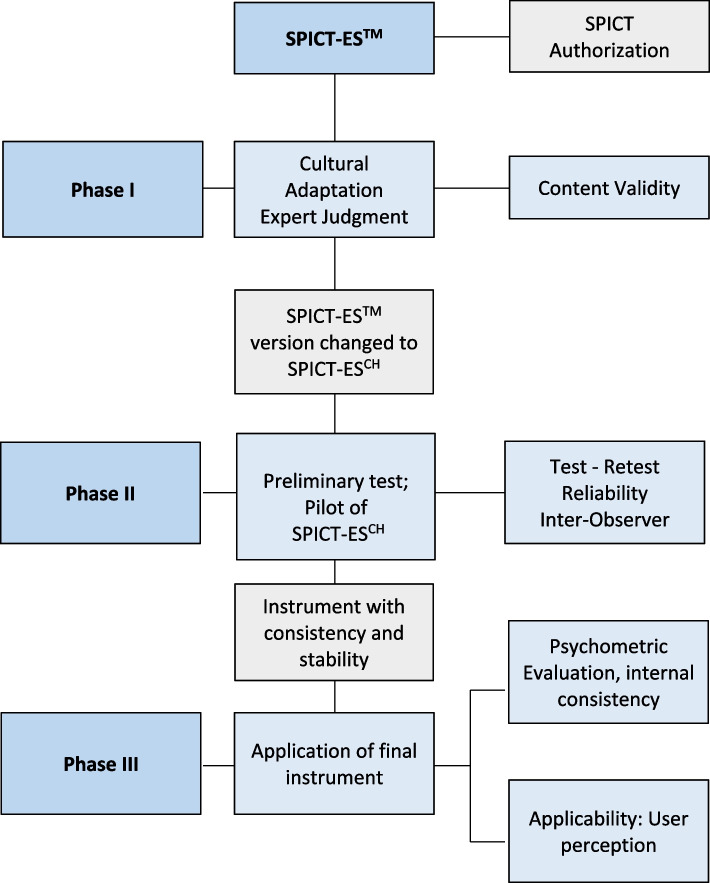
SPICT-ES^CH^ diagram

The study was done in three phases (Fig. 1) established according to recommendations from the literature [15, 16], with validation and transcultural adaptation to a Chilean context via expert judgment (Phaser I), a pilot study (Phase II) and applying the final instrument to evaluate internal consistency (Phase III).

The instrument was applied in 5 health centers established in different districts of the city of Santiago de Chile; 2 hospitals (1 public and 1 private) and 3 OA residences. The centers were included because of their different morbidity care coverage.

The sample and the target population included in each stage of the study was established according to the literature review. Likewise, the statistics used are described for each of the phases. The data was anonymized and coded in order to protect confidentiality.

Phase I was done through a concordance analysis with a panel of experts selected by eligibility criteria according to literature recommendations; study construct, professional competence, experience in clinical judgment, decision making, training and experience in their respective areas [15–17]. The expert panel consisted of 5 physicians and 6 nurses, professionals specializing in palliative care, internal medicine and/or geriatrics, with 3 years or more of clinical experience, who analyzed the linguistic, conceptual and cultural aspects of the instrument [16].

The judges' evaluation method is based on the analysis of experts until reaching an agreement for all the items according to the validity indicators of semantic and idiomatic equivalence; clarity, relevance and pertinence, according to the guidelines of cross-cultural adaptation (Beaton et al., 1993., 2000). Data collection was done by means of a 5 items Likert-type survey through Google Forms.

Content evaluation was performed using an online system and each item was assessed for its adequacy, clarity, consistency, and relevance [15, 18]. For this phase, the statistical methods used are based on the content validity index (IVC) and the content validity ratio (CVR) to determine the items of the instrument that are adequate and should be kept in the final version of the instrument and to establish the average validity of all items evaluated by the experts, respectively. According to the literature, the consensus for the study is established from a CVI of 0.80 and an RVC of 0.5, depending on the number of judges (Lawshe, 1975) [19]. Cronbach's alpha was used to strengthen the item correlation analysis between judges considering the number of variables in the instrument and Lawshe's coefficient to ensure that the concordance inter-judge is reliably positive, and that the preliminary version of the instrument complies with cross-cultural equivalence [20].

The instrument resulting from this stage was called SPICT-ES^CH^ and presented to the author for evaluation and comments.

Consistency and stability evaluations for the SPICT-ES^CH^, were done in the Phase II, via the test–retest method with a second application the instrument by the same observer and an interval of 10 days [15]. 30 patients were recruited [19], with the inclusion criteria being older than 65 and being entered in an OA home. Residents without cognitive deterioration signed informed consent, while for those with cognitive deterioration consent was obtained from their guardian. This phase was essential to verify conceptual and idiomatic equivalence, responsiveness and comprehension in the target population.

The instrument was applied by nurses who fulfilled the inclusion criteria for informed consent and who had participated in patient care. Nurses who had known the patient clinically for less than 3 days were excluded [10]. The statistic used for reliability between the two measurements was Pearson's correlation coefficient [15]. This phase was used to submit the SPICT-ES^CH^ to the final test and evaluate psychometric reliability characteristics.

Finally, Phase III evaluated reliability of SPICT-ES^CH^ to determine test measures what it is supposed to measure and the degree of correlation among the items [14, 21, 22]. The participants were recruited from 2 hospital centers, one public and one private, and from 3 OA homes from urban areas of Santiago, Chile, with the same eligibility criteria as in Phase II. 15 clinical nurses participated after signing informed consent and receiving training about the objectives and application of the SPICT-ES^CH^. To calculate sample size, we followed the recommendations of Nunnally regarding the number of items considering 30% non-answered [10].

For the statistics, was used Cronbach's α now with internal consistency perspective (value over 0.7) and the analysis of the relation between the global SPICT-ES^CH^ results and its items with Odd-Ratio (OR), *p*-value and its confidence interval (CI) at 95%, by a logistic regression model and coefficients plotted in “coef-plot”.

Application of the SPICT-ES^CH^ was also evaluated in terms of feasibility; application time, item pertinence, comprehension, clarity and interpretation of the scoring via a self-applied survey among the clinical nurses.

## Results

In the cultural adaptation model, all the items of the instrument were conserved and some semantic modifications were made. The analysis showed 3 items with a concordance below 0.80; *"caregiver overload", "significant weight loss" and "functional impairment due to cancer progression".* These underwent three further rounds of expert evaluation, introducing the suggested modifications (Table 1) until inter-judge concordance by index validity, ratio validity and an α of 0.93; 0.97 and 0.87, respectively. The analysis using Lawshe's coefficient showed an acceptability of the instrument of 84%.

**Table 1:** Semantic changes phase I, expert judgment for content validity

ORIGINAL QUESTION (SPICT-ES™, Fachado A, et al., 2018)	EXPERTS’ MODIFICATION (SPICT-ES^CH^)
“The caregiver for the patient needs more help and support”	“The caregiver for the ill person needs more help and support due to overload affecting various personal spheres (physical, psychosocial, professional and/or spiritual)”
“The person has had significant weight loss during the last few months, or remains underweight”	“The person has had significant unintentional weight loss greater than 10% of total weight in the last 6 months, or remains underweight”
“Functional deterioration due to oncological disease progression”	“Deterioration and general decrease of physical functions due to oncological disease progression”

**Fig. 2:**
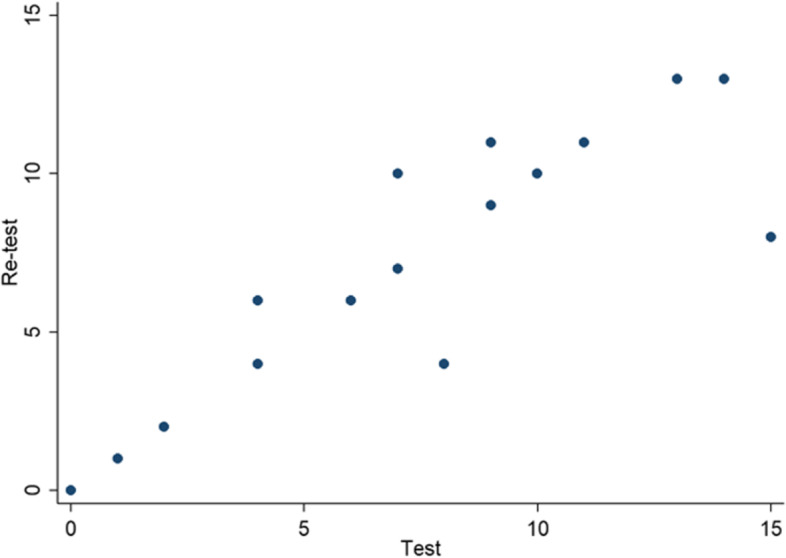
Analysis via correlation degree between measurements T1 and T2

The pilot study for the SPICT-ES^CH^ instrument showed a correlation with a Pearson coefficient (ρ) of 0.9167 (valor-*p* < 0.0001) revealing a linear relation between the two instrument measurements, consistency over time and a positive correlation between items (Fig. 2).

In the third phase, we analyzed 292 instruments after application. 66% were from females, with an average age of 79.4 and a SPICT-ES^CH^ positivity of 53.4% (Table 2) with a higher prevalence of cardiovascular disease (38%) compared to oncological disease (14%). Regarding comorbidity, 61% of patients presented 2 or more chronic diseases, with arterial hypertension and diabetes mellitus standing out (Table 3).

**Table 2:** Sociodemographic distribution and SPICT-ES^CH^, Chile 2022

Variable		n/mean^a^	%/SD^b^
SPICT-ES^CH^	Negative	136	46.58
	Positive	156	53.42
Sex	Female	188	64.38
	Male	104	35.61
Age		79.43	11.03

^a^Qualitative variables are described by absolute frequencies, while quantitative variables are described by averages

^b^Standard deviation (SD): Qualitative variables are described by relative frequencies and quantitative variables by standard deviation

**Table 3:** Patient morbidity prevalence SPICT-ES^CH^, Chile 2022

Pathology	%
2 or more Chronic Diseases	61
Cardiovascular Disease^a^	38
Cancer	14
Other pathologies^b^	12
Fragility/Dementia	11
Respiratory Disease	10
Fractures^c^	6
Renal Disease	3
Digestive Disease	1

^a^Heart Failure, Cardiopathy, Arrhythmia, ACV

^b^Dyslipidemia, Hypothyroidism, Depression, Sarcopenia, Obesity, Underweight, Urinary tract infection, Parkinson’s, Anemia

^c^Fracturing of hip, femur, or humerus

In the statistical analysis item 23 was eliminated, as it presented no variation and remained constant among all participants. According to the logistical model presented, the ORs were obtained for each question, along with their *p*-values and CI. Table 4 presents the highest OR values.

**Table 4:** Logistical model SPICT-ES^CH^ positive, Chile 2022

Item	OR	*p*-value	LI CI95%	LS CI95%
p2	30.81	< 0.001	16.14	58.85
p8	71.11	< 0.001	9.67	522.74
p9	43.52	< 0.001	5.88	321.91
p11	32.75	< 0.001	11.54	92.95
p12	48.77	< 0.001	11.65	204.18

The ORs obtained show that the items predict the positivity of the SPICT-ES^CH^. However, questions 20, 27 and 28 do not fulfill said capacity. Questions 22, 23 and 24 obtain an OR equal to 1, since when these questions are answered positively, there are no cases of negative SPICT-ES^CH^.

**Fig. 3:**
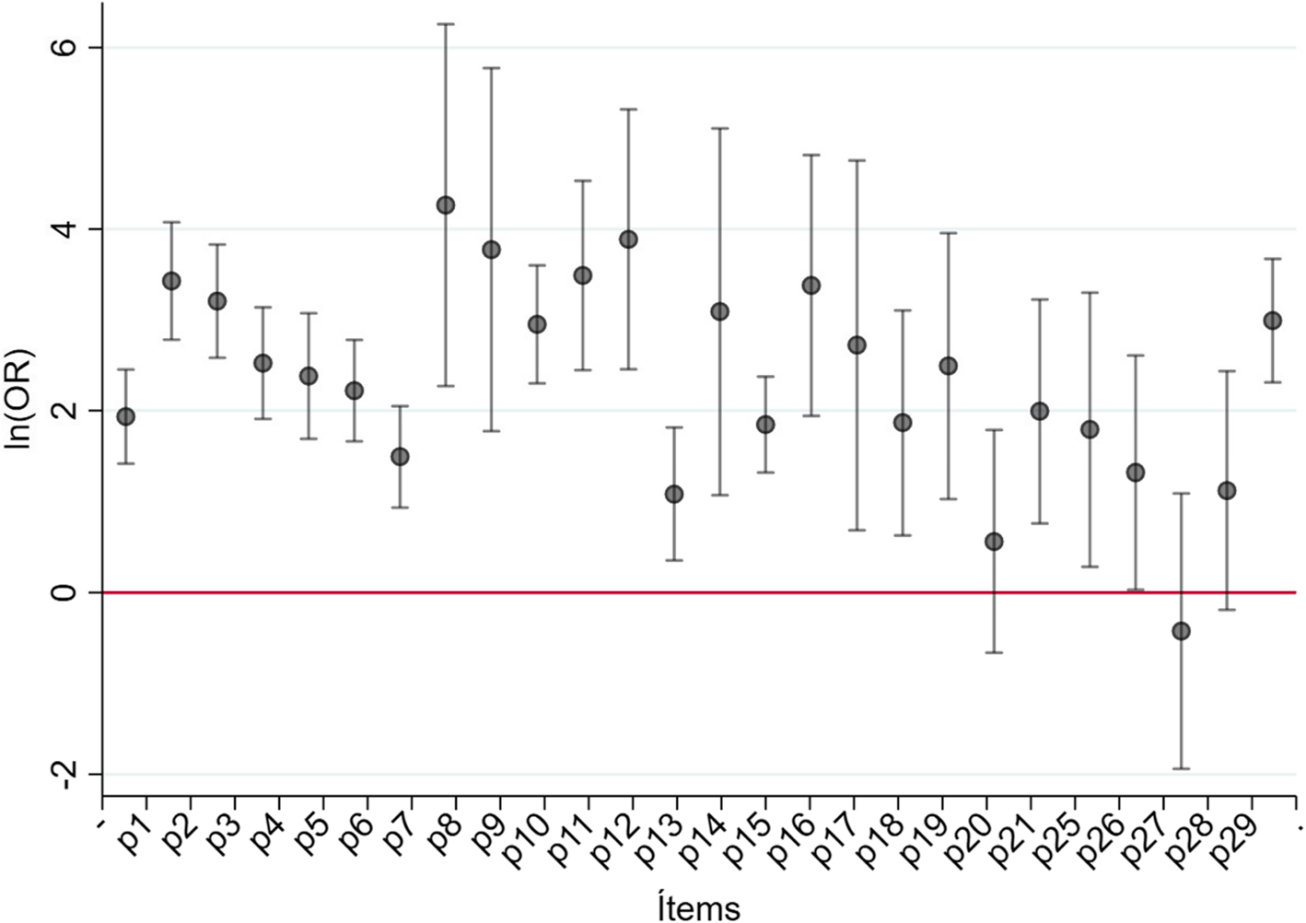
Analysis via degree of correlation between measurements T1 and T2

With the estimated OR, applying the natural logarithm function, we can obtain the model coefficients via which the point value can be graphed along with their confidence intervals (Fig. 3). The consistency analysis determined a Cronbach's alpha of 0.8662.

After the three phases of the study, the feasibility evaluation indicated that 100% of nurses *strongly agree* on the survey indicators that “the language of the SPICT-ES^CH^ is comprehensible”, “the time required to apply it favors its use”, and “recognizing patients who need palliative care would help with therapeutic decisions”. 75% of the professionals *agree* that “the instrument is applicable to the type of patient I work with” and “consider it useful in a clinical environment”.

## Discussion

The Spanish version of the SPICT-ES™ has been culturally adapted to the Chilean OA population according to the methodological steps proposed [15]. We have also evaluated its psychometric properties as a reliable instrument.

The cultural adaptation process for the instrument showed a semantic equivalence with the SPICT-ES™ translated into Spanish by Fachado A et al., (2018), making it necessary to add some words/expressions in 3 items to more clearly describe the indicators, similar to what was done in other adaptations [23, 24]. Based on the concordance level, it is interpreted that SPICT-ES^CH^ corresponds to an instrument that gathers evidence of content validity, with clear items, coherent with the construct to be measured and feasible to be submitted to the following stages.

The results of the pilot test determined excellent reliability, with a value above 0.9 according to Carvajal et al., (2011), demonstrating stability with an almost perfect correlation between the two applications.

In the final application of the SPICT-ES^CH^, its positivity reached 53.4% within all applications, similar to reports from other studies in the geriatric population, with a cut-off of two general indicators and one clinical [25, 26].

Another finding was that the most frequently measured items to determine a positive SPICT-ES^CH^, were clinical indicators; “*deterioration and decrease of physical function”, “loss of ability to speak with little social interaction”* and *“too frail for oncological treatment”.* This contrasts with Afshar et al., (2020) who mainly describe general indicators, except for “*functional limitation or persistent irreversible deterioration”,* another prevalent general indicator in the results of this study. However, considering the scientific literature both indicators, clinical and general, must be complemented [9].

Statistical analysis by item showed that 23 of them showed statistical significance, and therefore a greater chance of a positive SPICT-ES^CH^. Similarly, the items measured the aspect to evaluate, contributing to the usefulness of this instrument for detecting palliative needs [25]. The internal consistency of 0.8662 confirmed correlation and intercorrelation between items.

The clinical indicators of *“severe peripheral artery disease”, “cirrhosis with one or more complications”, “persistent respiratory failure”* and *“chronic terminal illness with deteriorating health”,* were present in a very low percentage of the sample analyzed. Therefore, its capacity to predict a positive SPICT-ES^CH^ is insufficient in this sample. Meanwhile, the conditions *“liver transplant”* and *“respiratory failure requiring mechanical ventilation”* were not reported in the measurements.

The usefulness of a clinical instrument is also measured by its feasibility of application and its acceptance by professionals [9]. The nurses who participated in this study considered that the SPICT-ES^CH^ is easily applied in geriatric populations with an average time of 3 min, which decreased following familiarization. They recognized its usefulness for detecting patients who required a palliative approach [24]. Non-identification of PC needs can have undesirable effects for both patients and their families [27].

Applying the SPICT-ES^CH^ could help drive early palliative care planning [28], in patients with oncologic and non-oncologic disease, improving quality of life and the dying process in this population.

### Study limitations

We did not perform any criteria validation, since the beginning of this study had no similar instrument validated in Chile as a gold standard. Only nurses participated during the SPICT-ES^CH^ application phases, which may bias the final results. This study was only done at low- to medium-complexity health care levels, without evaluating OA in more severe conditions o specifically primary care, however, patients of different levels of health complexity and socioeconomic level were included to enrich the results of the study.

## Conclusion

There are currently few psychometric analysis studies of SPICT-ES versions in a numerous group of OA. The results of this study are therefore a contribution to recognizing this instrument as a reliable tool to support the decision for an early start to palliative care in geriatric populations, with both oncological and non-oncological diseases.

Validation of the SPICT-ES^CH^ may contribute as a clinical tool for the timely detection of OA requiring palliative care at different levels of health care. This study will determine the delivery of the SPICT-ES^CH^ to be used by health professionals in the screening of palliative support needs. In this context, the necessary effort will be made to include it in the palliative care program for oncologic and non-oncologic patients, strengthening the implementation of the universal PC law in Chile, with the purpose of providing better care during terminal illness.

## Data Availability

The data sets generated and analyzed during this study are not available to the public, because they are in a private database. However, they are available from the corresponding author upon reasonable request.

## Abbreviations

PC: Palliative Care
ACD: Advanced chronic Illness
SPICT: Supportive & Palliative Care Indicators tool
SPICT-ES^CH^: Supportive & Palliative Care Indicators tool, Spanish version, Chile
CI: Confidence Interval
OR: Odd ratio
OA: Older adults
IVC: Content validity index
CVR: Content validity ratio
